# Neural Network-Based Prediction of Post-Operative Visual Outcomes Following Secondary Pediatric Intraocular Lens Implantation

**DOI:** 10.3390/children12101413

**Published:** 2025-10-20

**Authors:** Andrew Farah, Raheem Remtulla, Robert K. Koenekoop

**Affiliations:** 1 Faculty of Medicine and Health Sciences, McGill University, Montreal, QC H3G 2M1, Canada; andrew.farah@mail.mcgill.ca; 2 Department of Ophthalmology & Visual Sciences, Faculty of Medicine and Health Sciences, McGill University, Montreal, QC H3G 2M1, Canada; raheem.remtulla@mail.mcgill.ca; 3 McGill University Health Center Research Institute, Montreal, QC H3G 2M1, Canada; 4 Departments of Pediatric Surgery, Human Genetics, and Adult Ophthalmology, McGill University Health Center, Montreal, QC H3G 2M1, Canada

**Keywords:** congenital cataract, pediatric ophthalmology, intraocular lens, artificial intelligence, machine learning, neural networks, visual outcomes

## Abstract

**Highlights:**

**What are the main findings?**

- A proof-of-concept neural network model was developed to predict visual outcomes after secondary intraocular lens (IOL) implantation in children with congenital cataracts.
- The model demonstrated encouraging predictive performance across training, validation, and test sets, suggesting feasibility despite the limited dataset.

**What is the implication of the main finding?**

- This work underscores the potential of machine learning to support clinical decision-making for secondary IOL implantation, an area currently lacking predictive tools.
- Broader, multi-center datasets and models restricted to preoperative variables will be essential to validate and translate this approach into clinical practice.

**Abstract:**

Objectives: To develop a proof-of-concept machine learning (ML) neural network model to predict post-operative visual outcomes in children with congenital cataracts undergoing intraocular lens (IOL) implantation, thereby guiding the optimal timing for IOL insertion. Determining the ideal timing and predicting outcomes for IOL implantation in children remains clinically complex due to variability in eye development and measurement accuracy. Methods: Retrospective analysis using a publicly available dataset from 110 children diagnosed with congenital cataracts, who underwent IOL implantation at the Eye and ENT Hospital of Fudan University. A neural network model with a hidden layer of 10 nodes was developed in MATLAB 2024a using the scaled conjugate gradient algorithm. Input variables included demographic and clinical features; the target was achieving visual acuity greater than 20/40. Performance metrics were evaluated using cross-entropy loss, sensitivity, specificity, and accuracy. Results: Training completed after 14 epochs with the test set reaching the highest performance metrics: 88.2% accuracy, 88.9% sensitivity, and 87.5% specificity. ROC curve analysis showed AUC values of 0.942 (training), 0.920 (validation), 0.885 (test), and 0.917 (overall). Conclusions: The neural network effectively predicted post-operative visual outcomes, offering potential clinical utility in guiding IOL implantation decisions. Despite limitations in dataset diversity, this study lays the foundation for future development of personalized strategies in pediatric cataract care.

## 1. Introduction

One of the most significant treatable causes of childhood blindness worldwide is congenital cataract [1,2]. This form of cataract is identified by an opacity of the crystalline lens and often diagnosed at birth or during the initial months of childhood [1,2]. While the majority of congenital cataract cases are idiopathic, it is associated with other medical conditions such as Down syndrome and Lowe syndrome or can develop due to certain pathogens (toxoplasmosis, rubella, cytomegalovirus, herpes and syphilis) during pregnancy [1].

Treatment for cataract in adults consists of removing the opacified lens followed by an intraocular lens (IOL) implantation. Unfortunately, various dilemmas arise with the usage of IOL in children with congenital cataract, and this procedure remains controversial [3]. Several studies investigating the optimal timing for cataract surgery in children conclude that it depends on balancing the risks of early surgery, which increases the risk of glaucoma, against delayed surgery, which can raise the risk of strabismus and deprivation amblyopia [4,5,6,7]. Furthermore, preoperative examinations and measurement accuracy can be complicated on sedated patients and selecting the appropriate IOL is complex due to significant postoperative eye growth, corneal curvature variability and limited cooperation [8,9,10]. Pediatric cataract patients experience greater imprecision and higher prediction errors in IOL power calculation compared to adults, requiring the use of different formulas based on age and axial length [8,9,11]. As a result, the challenge in accurately predicting postoperative axial lengths often leads to refractive errors like significant hyperopia or myopia [9,10,12].

In light of these challenges, traditional approaches may fall short in consistently guiding individualized choices in secondary IOL implantation. Recently, ML, a branch of artificial intelligence that develops predictive models by learning from large datasets, has emerged as a powerful tool for extracting clinical insights tailored to specific patient contexts [13,14,15,16]. ML has already shown promising results, for instance, models predicting postoperative refractive outcomes in adult cataract surgery have achieved accuracy comparable to conventional IOL power calculation formulas [16,17]. For example, they have shown strong performance in detecting and staging retinopathy of prematurity and other pediatric fundus diseases, with reproducible assessments where clinical judgment is variable [14,18,19]. In congenital cataract, Long et al. have developed CC-Guardian, an AI system combining Bayesian and ML methods to guide timing and treatment planning, while Zhang et al. created a prognostic survival model for long-term VA outcomes in bilateral cases [20,21]. Collectively, these studies illustrate the growing promise of AI for pediatric ophthalmology, although efforts directly addressing secondary IOL decision-making remain sparse.

Driven by these advancements and the complexity of pediatric cataract management, we developed a proof-of-concept ML model to predict postoperative visual potential in children undergoing secondary IOL implantation, where preoperative visual acuity cannot be reliably measured.

## 2. Materials and Methods

Our study involved a publicly available dataset by Rong et al. providing comprehensive information regarding a cohort of 110 children diagnosed with congenital cataracts who underwent IOL implantations [22]. Data was collected at the Eye and ENT Hospital of Fudan University, Shanghai between 1 January 2001 and 31 December 2007. The original study was conducted in accordance with the Declaration of Helsinki and received prospective approval from the hospital’s ethics committee, with written informed consent being obtained from the guardians of all participating children. Certain exclusion criteria were used for the development of this dataset including patients with conditions of traumatic cataracts, retinopathy of prematurity, persistent fetal vasculature, congenital glaucoma, microphthalmos, along with other systemic diseases. Various procedures were performed by an experienced surgeon on sedated patients including anterior capsulorhexis, cataract irrigation and aspiration, posterior capsulectomy and anterior vitrectomy. All cases were followed with secondary implantation of a three-piece hydrophobic acrylic foldable IOL and standard postoperative care. Moreover, the follow-up regimen for these patients was rigorous with frequent ophthalmological assessments and meticulously tracked long-term visual outcomes. Baseline characteristics of this cohort have been reported in detail by Rong et al. In brief, the mean age at cataract extraction was 7.45 ± 4.73 months (bilateral) and 8.32 ± 4.91 months (unilateral), while the mean age at secondary IOL implantation was 46.64 ± 29.37 and 33.82 ± 16.43 months, respectively. The mean follow-up period after IOL implantation was approximately 95 months, with a range spanning from 65 to 166 months [22].

**Figure 1:**
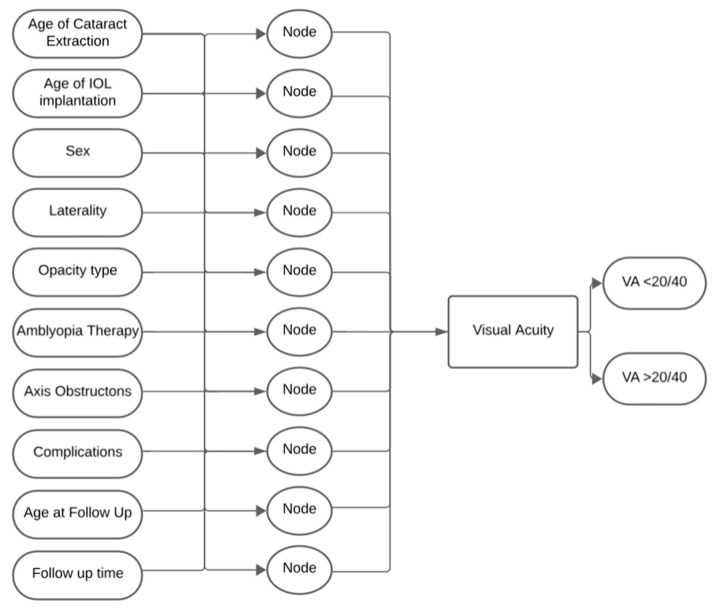
Neural Network Process Diagram.

Using the updated version of MATLAB 2024a released on 4 April 2024, by MathWorks, we developed a feed-forward single-layer neural network model based on the neural network starter tool. The hidden layer in our network architecture comprised 10 nodes corresponding to the input factors provided in the dataset. These factors included biological sex, age at cataract extraction, age at IOL implantation, age at last follow-up, laterality, follow-up time, opacity type, amblyopia therapy, pre- and post-visual axis obscuration and post-operative complications. We employed a binary classification for the targeted output indicating whether patient visual acuity (VA) was at least 20/40 as of the latest follow-up. At the latest follow-up, 17.7% of eyes (23/130) achieved a best corrected VA (BCVA) better than 20/40, including 25.6% of bilateral cases (22/86) and 2.3% of unilateral cases (1/44). The process diagram of our neural network training is shown in Figure 1.

**Figure 2:**
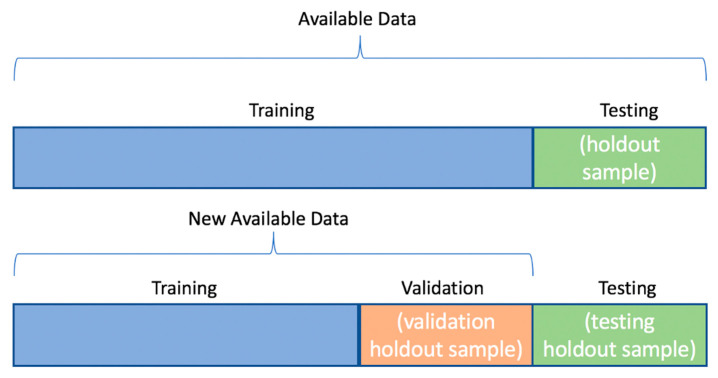
Data Partition for Development of Neural Network.

Next, we randomly divided the available data into three subsets: 75% for training (*n* = 82), 10% for validation (*n* = 11), and the remaining 15% for testing (*n* = 17) (Figure 2). This allocation was selected given the relatively small sample size, to maximize the proportion of data available for training while still retaining sufficient cases for both validation and independent testing. We also enabled early stopping, which monitored validation performance and terminated training when improvement plateaued, thereby reducing overfitting. With our data and variables prepared, we opted to train our neural network with the scaled conjugate gradient algorithm. This algorithm is known for its efficiency, suitability and faster convergence for medium-sized feedforward networks while remaining fully automated [23]. To ensure accurate classification and monitor our model’s performance, we used the cross-entropy loss function, an effective tool for binary classification that computes the discrepancy between predicted probability distributions and ground truth.

Our statistical analysis consisted of benchmarking model performance through usage of cross-entropy as our foundational metric. Then, once the optimal validation performance was determined, we calculated the associated sensitivity, specificity, and accuracy for each of the randomized subsets of data, as well as the overall metrics across all the data combined.

## 3. Results

### 3.1. Cross-Entropy Loss and Training

**Figure 3:**
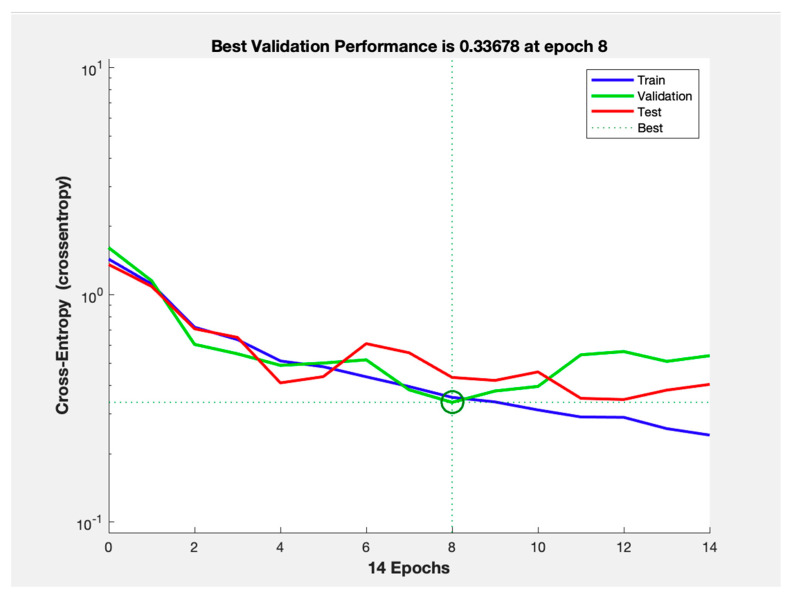
Cross-entropy Loss Performance Per Training, Validation and Test Datasets.

We deployed our model while closely tracking training, validation and test performance across 14 epochs, as illustrated in Figure 3. The plot follows the cross-entropy loss on a logarithmic scale for their respective data subsets. The best validation performance was achieved at epoch 8 with an approximate cross-entropy value of 0.33678. Subsequently, signs of overfitting may have emerged, as the training loss continued to decline slightly despite the validation loss remaining stable. The test subset exhibited a similar trend with consistent performance when compared to the validation loss.

### 3.2. Accuracy, Sensitivity, and Specificity by Dataset

While remaining at the best validation performance based on our cross-entropy loss tracking, we evaluated our model across the training, validation, and test datasets, with results summarized in Table 1. The training set demonstrated a specificity of 85.7%, sensitivity of 85.0%, and accuracy of 85.4%. In the validation set, specificity slightly decreased to 83.3%, with sensitivity dropping to 80.0% and accuracy to 81.8%. The test set exhibited the strongest performance, with the highest specificity, sensitivity and accuracy at 87.5%, 88.9% and 88.2%, respectively. Overall, the network maintained a specificity of 85.7%, a sensitivity of 85.2%, and an accuracy of 85.5%.

### 3.3. Receiver Operating Characteristic (ROC) Analysis

**Figure 4:**
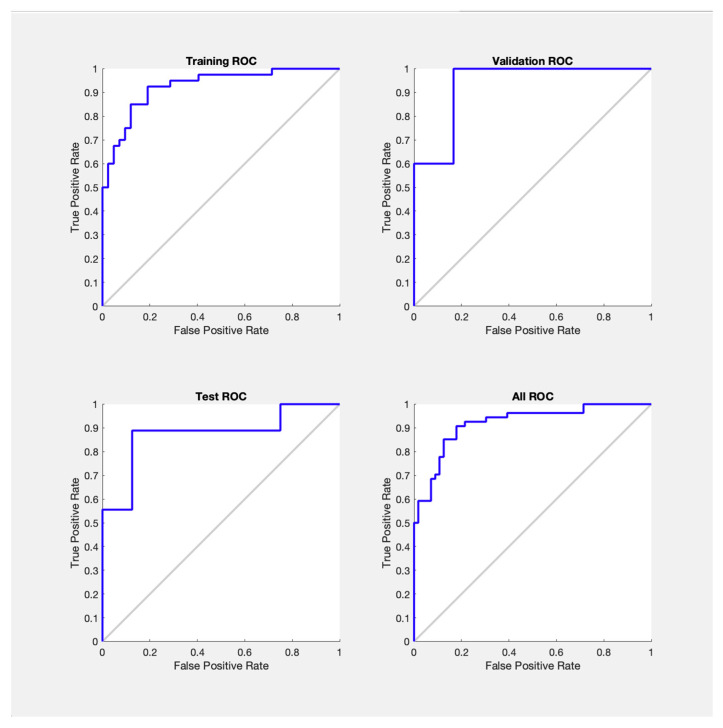
Receiver Operating Characteristic Curves Per Dataset.

The ROC curves for the model’s performance on the training validation, test and combined datasets are presented in Figure 4. Each subplot represents the ROC curve for one of these datasets, plotting the trade-off between the true positive rate against the false positive rate. The area under the curve values were calculated giving 0.942 for the training set, followed by 0.920 in the validation set, 0.885 in the test set, and an overall AUC of 0.917.

## 4. Discussion

Current clinical practice regarding the timing of IOL insertion following congenital cataract surgery has significant variability. While there is a general consensus about the safety of IOL use for older children, there are no official guidelines in the case of children during the first year of life [24,25]. In fact, due to the inability for IOL to change in power following rapid eye growth in early childhood, aiming for emmetropia will only leave the patient highly myopic in adulthood [12,24]. As a result, IOL implantation may be delayed, and aphakic children are typically corrected with contact lenses or glasses while managing amblyopia until the secondary IOL implantation [25,26,27]. While some studies have reported successful postoperative outcomes of secondary IOL insertion in children as young as two years old, others advocate for delaying the procedure until the child reaches elementary school age to achieve a more predictable refractive outcome [28,29]. This is where ML neural networks can serve as powerful tools that enhance decision-making in an area of treatment that has traditionally been uncertain. By inputting patient-specific information into a neural network algorithm, it becomes possible to offer a data-driven and standardized method to predict post-operative visual outcomes. Previous ML studies in adult cataract surgery have demonstrated this with strong predictive performance in postoperative refraction, while pediatric applications have focused on primary IOLs and bilateral congenital cases [16,17,20,21]. In contrast, our work extends the application of ML to secondary IOL implantation, a domain lacking predictive models, and offers a framework for identifying optimal timing by balancing early visual rehabilitation with long-term refractive stability.

We evaluated our model’s performance based on whether the post-operative BCVA was better or worse than 20/40 at long-term follow-up, since preoperative VA in infants with dense congenital cataracts was not systematically measurable. In Canada, a standard driver’s license requires VA not worse than 20/50 and commercial licenses demand a minimum of 20/30 [30]. Meanwhile, in the United States, all but three states have the minimum BCVA at 20/40 [31]. In fact, Canadian sources commonly define visual impairment as having a visual acuity of less than 20/40 [32,33,34]. Importantly, low vision is often defined as BCVA worse than 20/40 that cannot be improved with glasses, contact lenses, or surgery, which underscores the importance of long-term functional vision beyond mere refractive correction [35]. Even mild vision loss significantly worsens quality of life—a burden often underestimated—with affected individuals willing to trade years of life for perfect vision [36,37]. As such, patients with an uncorrected VA worse than 20/40 would generally be advised to use corrective spectacles or other visual aids. To better predict and address the common outcome of myopia, which is a primary concern in congenital cataract correction, we set the target VA for our neural network model closer to emmetropia [12]. Preoperative VA and axial length assessment have significant variability and are difficult to measure reliably in infants with dense congenital cataracts; their use was limited in our model development since these variables were not systematically collected in this cohort [9,10]. This further highlights the need to develop accurate predictive models for visual outcomes following secondary IOL insertion, to support optimal patient care and improve quality of life.

By leveraging a comprehensive and publicly available long-term dataset, we developed a proof-of-concept neural network model exhibiting high accuracy, sensitivity and specificity, providing a means to more accurately identify the optimal timing for secondary IOL insertion. Cross-entropy loss was pivotal in fine-tuning our model’s performance, allowing us to minimize the risks of overfitting by identifying the moment of optimal accuracy. Early stopping was used because the networks can theoretically run indefinitely without improving performance. Combined with the carefully divided dataset, we ensured a balanced approach that renders the model’s predictions to be both dependable and clinically relevant. One of our study’s strengths is the use of a multilayered algorithm integrating patient-specific factors across 10 nodes, paving the way for more standardized and personalized healthcare guidelines. This is further reinforced by the model’s high consistent predictive performance across all datasets, as demonstrated by the high AUC values and the excellent overall AUC [38]. The minimal drop in performance in AUC from training to testing is expected and showcases how the model generalizes well to unseen data with a minimal risk of overfitting. This consistency highlights the model’s excellent ability to distinguish between patients likely or unlikely to achieve favorable visual outcomes and strengthens its potential for clinical translation, which is often marked by uncertainty.

Our study offers valuable insights, yet it is not without certain limitations. The dataset was collected from a single center, with all surgeries performed by the same surgeon and IOL type, which limits applicability to settings with different populations, techniques and materials. Alternative designs, such as the Bag-in-the-Lens (BIL) and posterior optic capture (POC) approaches, have been shown to reduce posterior capsule opacification and may therefore influence long-term visual outcomes [39,40,41]. The limited sample size (110 patients) also restricted validation robustness, raising the risk of overfitting and required dichotomization of VA at >20/40 as more granular analyses would have lacked sufficient power to reliably assess the relative contribution of individual predictors. This threshold also reflects North American conventions and may not generalize globally. The relevance of our findings may also be affected by data collected between 2001 and 2007, when surgical techniques and postoperative care differed from current practice, though the long-term follow-up remains valuable for proof-of-concept purposes. External validation was not performed, as existing pediatric cataract datasets differ substantially in baseline characteristics, treatment protocols, and outcome measures [42,43]. For example, the Infant Aphakia Treatment Study focused on unilateral cataracts managed with contact lenses or primary IOLs, making it unsuitable for comparison with our secondary IOL cohort [44]. Likewise, the exclusion of comorbid cases may have introduced selection bias, reducing external validity. This study remains exploratory in nature, as our usage of post-operative predictors (e.g., complications, visual axis obscuration, follow-up duration, etc.) limits the direct use of the present model as a pre-operative clinical tool.

Future research should expand on this proof-of-concept by incorporating multi-institutional datasets that capture broader demographic and clinical diversity. The inclusion of different IOL types and implantation techniques, such as the BIL, optic capture and iris fixated lenses, will be essential for evaluating generalizability across surgical practices [39,45,46]. Prospective studies with updated surgical techniques, external validation, and additional patient parameters (e.g., axial length, variation in VA) could enhance accuracy and applicability. Methodological refinements such as k-fold cross-validation, benchmarking against simpler models (e.g., logistic regression, random forests), and feature importance analyses will aid in contextualizing the incremental benefit of machine learning models. Future cohorts should also incorporate calibration, bootstrapped confidence intervals, and decision-curve analysis to quantify uncertainty and clinical utility.

## 5. Conclusions

This proof-of-concept study underscores the significant promise of machine learning models in predicting post-operative visual outcomes for children with congenital cataracts undergoing secondary IOL implantation. Our neural network, trained on a robust and long-term dataset, effectively used the age of IOL implantation as a key variable to predict which patients would experience VA improvements greater than 20/40. While the present model is not yet suitable for clinical decision-making, these insights have the potential to become an invaluable tool in optimizing surgical timing and selecting appropriate IOL power to optimize patient care. Its performance requires confirmation in larger, multi-center cohorts, with models restricted to preoperative variables, external validation and benchmarking against simpler approaches. With these steps, this pilot study provides a foundation for future research aimed at the development of personalized treatment strategies in pediatric cataract care and eventual clinical translation.

## Figures and Tables

**Table 1:** Specificity, Sensitivity and Accuracy Per Dataset.

Performance Metrics	Training	Validation	Test	Total
Specificity	85.7%	83.3%	87.5%	85.7%
Sensitivity	85.0%	80.0%	88.9%	85.2%
Accuracy	85.4%	81.8%	88.2%	85.5%

## Data Availability

The dataset used in this study is publicly available and was originally provided by the authors of the source publication for research and educational use. It can be accessed at the following link: https://datadryad.org/stash/dataset/doi:10.5061/dryad.5t9d1 (accessed on 20 June 2024).

## Abbreviations

The following abbreviations are used in this manuscript:

IOL	Intraocular Lens
VA	Visual Acuity
ML	Machine Learning
AI	Artificial Intelligence
BCVA	Best Corrected Visual Acuity
ROC	Receiver Operating Characteristic Curve
AUC	Area Under the Curve
